# Supplementary material for: Going rogue: what scientists can learn about Twitter communication from “alt” government accounts
Source: PeerJ. 2021 Nov 2;9:e12407. doi: 10.7717/peerj.12407 (PMC8570174; doi:10.7717/peerj.12407)
Supplement: Supplemental Information 1 [file peerj-09-12407-s001.pdf]

Appendix 1: All accounts associated with U.S. federal agencies or organizations, officially or unofficially, with total number of tweets (including retweets), followings, followers, and likes.

Agency		Account	Tweets	Following	Followers	Likes
National Park Service		***@NatlParkService***	*7870*	*2699*	*433856*	*3853*
		@NotAltWorld	*502*	*229*	*1328031*	*60*
		@realALTNPS	19	1	28470	0
		@AltNatParkSer	*156*	*60*	*88820*	*20*
		@ALTUSNPS	362	194	20970	3763
		@aItnps	560	75	10692	0
		@Rogue_NPS	89	53	7314	23
	Alaska Region	**@AlaskaNPS**	3198	543	36578	387
		@AltNPSAlaska	688	94	5571	974
	Badlands	***@BadlandsNPS***	*3793*	*1695*	*234566*	*14518*
		@AltBadlandsPark	117	100	28322	25
		@AltBadlandsNPS	542	2812	29677	2136
		@BadHombreNPS	*1193*	*324*	*208629*	*10.6*
		@BadHombresNPS	69	378	12988	435
		@BadIandsNPSFans	33	3	111382	7
		@badasslandsUSA	153	476	1579	133
		@BadlandsGonWild	60	0	4644	25
	Big Bend	**@BigBendNPS**	542	118	6706	483
		@AltBigBendNP	88	103	676	320
	Biscayne	**@BiscayneNPS**	2259	274	12445	641
		@AltBiscayneNPS	405	278	1612	274
	Crater Lake	**@CraterLakeNPS**	2183	127	29031	0
		@AltCraterLakeNP	81	104	17470	16
		@AltCraterLake	5	33	698	5
	Dry Tortugas	**@DryTortugasNPS**	739	389	13103	895
		@AltTortugasNPS	178	40	1721	31
	Everglades	**@EvergladesNPS**	1953	318	21421	1378
		@AltEverglades	157	522	1958	1
	Glacier	**@glaciernps**	2920	259	193464	766
		@AltGlacierNPS	224	74	39095	43
		@GlacierNPSAlt	237	681	28310	184
	Homestead Mnmt	**@HomesteadNM**	2966	679	11750	84
		@AltHomesteadMon	1292	231	6335	428
	Hoover Historical Site	**@HooverNPS**	329	328	10432	11
		@AltHooverNPS	811	62	20699	277
	Hot Springs	**@VisitHotSprings**	1787	314	3584	215
		@TheRogueArkie	513	91	31231	230

	Lassen	**@LassenNPS**	917	25	26081	3
		@AltLassenNPS	148	1134	35647	474
	Mt Rushmore	**@MountRushmoreNM**	738	799	1922	270
		@AltRushmore	66	32	1632	50
	Muir Woods	**@MuirWoodsNPS**	1612	469	5186	507
		@altMuirWoodsNPS	175	241	19626	50
	Olympic	**@OlympicNP**	740	334	12025	740
		@olympicRogueNPS	375	237	34934	983
	Pullman Mnmt	**N/A**				
		@AltPullMonument	415	483	254	211
	Rainier	**@MountRainierNPS**	3709	7	27923	727
		@AltMtRainier	1602	299	68139	6461
	Rocky Mnt	**@RockyNPS**	12204	69	27765	7
		@AltRockyNPS	347	73	40225	158
	Saguaro	**@SaguaroNPS**	1879	39	21972	104
		@altSaguaroNPS	43	1160	734	29
	Volcanoes	**@Volcanoes_NPS**	667	547	9985	694
		@AltHVNP	1749	595	34411	4324
	Yellowstone	**@YellowstoneNPS**	3689	377	499277	1890
		@AltYelloNatPark	5443	156	53187	5231
	Yosemite	**@YosemiteNPS**	4654	12	330647	78
		@AltYosemite	78	125	70371	464
		@YosemiteUprisin	178	213	18795	253
	Zion	**@ZionNPS**	2356	777	83794	2553
		@alt_nps_zion	33	8	637	5
Bureau of Land Management		**@BLMNational**	9696	1125	22681	13014
		@blm_alt	140	160	11902	69
		@AltBLM	163	27	1010	84
Centers for Disease Control and Prevention		**@CDCgov**	18316	271	781394	266
		@Alt_CDC	219	217	215698	96
		@viralCDC	228	56	21908	463
Department of the Interior		**@Interior**	15589	229086	2924739	2637
		@InteriorAlt	1581	648	3502	2024
Environmental Protection Agency		***@EPA***	*13415*	*677*	*504072*	*6*
		@ActualEPAFacts	3595	110	292649	2158
		@altUSEPA	*4629*	*84*	*388174*	*7251*
		@ungaggedEPA	564	836	102682	4357
		@EPAWouldSay	285	107	25294	266
		@RogueEPAstaff	2718	959	3782	1666
		@RogueUSEPA	168	31	9853	35
		@Uncensored_EPA	126	73	1299	24
		@TheAltEPA	721	514	10787	1064

	@RealAltUSEPA	2493	949	1360	2055
Food and Drug Administration	**@US_FDA**	6628	140	160910	168
	@alt_fda	802	270	197508	153
	@rogueUSFDA	87	22	2123	101
Health and Human Services	**@HHSGov**	13387	244	664928	130
	@AltHHS	261	207	216669	87
	@RogueHHS	4084	355	1038	594
National Aeronautics and Space Administration	**@NASA**	46448	263	22592355	2381
	@ResistanceNASA	197	530	51758	241
	@RogueNASA	1335	264	899606	1093
	@Alt_NASA	536	86	207329	308
	@TheAltNasa	195	676	709	4
	@aItNASA	23	65	4068	0
National Institutes of Health	**@NIH**	7087	256	756915	578
	@Alt_NIH	75	38	219232	13
	@AltNIH	16	67	2443	3
National Institute of Standards and Technology	**@usnistgov**	8380	281	34273	413
	@alt_NIST	209	27	2011	12
National Oceanic and Atmospheric Administration	***@NOAA***	*10063*	*117*	*826777*	*16*
	@RogueNOAA	41	17	53415	0
	@altNOAA	*2208*	*250*	*146434*	*784*
	@noaagov	31	31	27175	3
National Marine Fisheries Service	**@NOAAFisheries**	5858	409	37076	32
	@AltNMFS	350	33	868	9
National Science Foundation	**@NSF**	16380	98	1034752	3561
	@Rogue_NSF	146	110	9088	13
	@ALT_NSF	28	84	2457	88
	@AltNatSciFdn	0	34	10	0
Smithsonian Institution	**@smithsonian**	16902	207	2530667	1023
	@AltSmithsonian	1157	285	24283	1065
Arctic Research Commission	**@US_ARC**	6970	13	4114	
	@AltUS_ARC	139	33	57490	11
Board on Geographic Names	**N/A**	**N/A**	**N/A**	**N/A**	**N/A**
	@Rogue_USBGN	0	0	9	0
	@Rogue_Sci	122	46	55067	29
Department of Agriculture	***@USDA***	*12101*	*2027*	*535671*	*95*
	@USDA_alt	120	99	10835	45
	@altusda	*1634*	*25*	*258361*	*5047*
Agricultural Research Service	**@USDA_ARS**	3022	191	13655	225
	@AltUSDA_ARS	5046	513	19568	2477
Forest Service	***@forestservice***	*21178*	*1901*	*123691*	*502*

	@AltForestServ	*282*	*188*	*338555*	*83*
Fish and Wildlife Service	***@USFWS***	*13463*	*48967*	*183653*	*28456*
	@AltUSFWSRefuge	744	56	41411	645
	@AltUSFWS	*5530*	*504*	*74710*	*798*
	@AlternateUSFW	684	357	19615	859
Geologic Survey	**@USGS**	13850	90	653303	871
	@Alt_USGS	94	226	333	129
Combination Alt Accounts	@altNPSandEPA	69	51	32608	31
	@NastyWomenofNPS	1493	521	12669	1766
	@AngryNatlPark	1767	190	29395	567
	@ClimateNPS	275	232	29796	289
	@BowlingGreenNPS	133	92	1838	470
	@YellerstoneNPS	414	265	6480	395
	@MordorNPS	384	352	16112	649
	@SubversivRanger	3234	2656	1123	601
Non-Science					
AmeriCorps	**@AmeriCorps**	8304	802	55962	6315
	@AltAmeriCorps	237	987	1147	363
American Library Association	**@ALALibrary**	16360	6299	144944	2037
	@rogue_ALA	233	363	7326	1716
Bureau of Alcohol, Tobacco, Firearms and Explosives	**@ATFHQ**	5315	3505	37495	920
	@Rogue_ATF	1	23	502	0
Armed Forces	**@USArmy**	26981	513	1041092	1251
	@AltArmedForces	110	25	271	11
Department of Energy	**@ENERGY**	13417	505	638029	2254
	@realUSDOE	43	52	19308	32
	@AltDofEnergy	1	2	17377	0
Department of Labor	**@USDOL**	19261	542	373129	2579
	@alt_labor	5521	781	62308	10649
Department of Education	**@usedgov**	20209	170	1171535	1974
	@RogueED	3364	326	9722	3246
	@Alt_DeptofED	1202	93	131656	1046
	@AltDptEducation	3828	156	28547	4786
Drug Information Association	**@DefenseIntel**	1397	82	102357	368
	@AltDIA	1162	57	26577	3244
Department of Transportation	**@USDOT**	5290	1198	159599	686
	@altDMV	156	70	2319	43
Department of Defense	**@DeptofDefense**	17196	980	4003949	1319
	@Rogue_DoD	222	181	32787	146
Department of Homeland Security	**@DHSgov**	11834	368	1261077	176

	@AltHomelandSec	2050	109	65910	369
Department of Justice	**@TheJusticeDept**	5717	218	1292043	0
	@AltUSDOJ	360	199	13252	197
	@AltBJS	218	4456	1777	261
Department of State	**@StateDept**	47543	401	4075264	15
	@AltStateDpt	1093	35	177743	465
Department of the Treasury	**@USTreasury**	5486	247	719802	210
	@alt_treasury	5	30	67283	0
Department of Veterans Affairs	**@DeptVetAffairs**	18608	879	505505	1007
	@AltDept_of_VA	1210	266	3132	589
Federal Bureau of Investigation	**@FBI**	9488	2189	1895029	0
	@altFBI	767	87	59813	35
Federal Election Commission	**@FEC**	977	1	8989	4
	@alt_fec	953	101	31284	504
Federal Emergency Management Agency	**@fema**	12121	638	581886	1745
	@rogueFEMA	542	245	41063	371
House Committee on Science, Space, and Technology	**@HouseScience**	2447	319	181793	177
	@altHouseScience	2222	113	2618	50
Department of Housing and Urban Development	**@HUDgov**	7555	348	240477	61
	@AltUSDeptHUD	154	77	41602	44
	@HUDFacts	76	1503	11737	44
National Education Association	**@NEAarts**	68045	769	88669	13076
	@Alt_NEArts	779	780	9158	616
National Endowment for the Humanities	**@NEHgov**	14124	789	25612	4070
	@RogueNEH	82	256	8561	126
	@alt_neh	40	270	2603	5
Peace Corps	**@PeaceCorps**	13847	382	1276812	2031
	@AltPeaceCorps	93	77	1776	14
Railroad Retirement Board	**N/A**	**N/A**	**N/A**	**N/A**	**N/A**
	@RogueRRB	1059	99	6927	7
Agency for International Development	**@USAID**	23863	657	629487	1258
	@Alt_USAID	113	42	2831	12
Citizenship and Immigration Services	**@USCIS**	9966	109	94563	93
	@ALT_USCIS	11733	192	32673	1
Refugee, Asylum and International Operations Directorate	**N/A**	**N/A**	**N/A**	**N/A**	**N/A**
	@AltRAIO	564	355	3553	35
	@ALT_RAIO_USCIS	4	640	135	0
The White House	**@WhiteHouse**	356	11	14136901	8
	@RoguePOTUSStaff	2242	44	847809	231

	@alt_Trump_WH	136	77	326	1
	@AltWhitehouseIT	1041	207	2273	300
	@WHLeaks	3	37	148	0
	@AltWhtHouse	88	58	260	0
	@white_house_alt	31	31	30	30
	@Alt_WhiteHouse_	74	34	39	0
	@RogueSNRadvisor	421	39	67535	22
	@AltEEOB	2182	192	8023	103
Press Secretary	**@PressSec**	343	39	1776826	41
	@AltUSPressSec	8830	659	16945	506
